# Registered nurses’ experiences regarding operational factors influencing the implementation of HIV care services in the mobile health clinics of eThekwini Municipality in KwaZulu-Natal

**DOI:** 10.1186/s12912-024-01764-9

**Published:** 2024-02-06

**Authors:** Silingene Joyce Ngcobo, Lufuno Makhado, Leepile Alfred Sehularo

**Affiliations:** 1 Lifestyle Diseases Research Focus Area, Faculty of Health Sciences, North-West University, Mmabatho, South Africa; 2 School of Nursing and Public Health, College Health Sciences, University of KwaZulu Natal, Durban, South Africa; 3 Department of Public Health, Faculty of Health Sciences, University of Venda, Thohoyandou, South Africa

**Keywords:** Mobile health clinics, NIMART implementation, Nurses, Operational factors, Community-based clinics, HIV care, Decentralised HIV care

## Abstract

**Background:**

Registered nurses working in the mobile health clinics (MHCs) play an important role in enabling HIV care access to populations in remote areas through Nurse Initiated Antiretroviral Therapy program (NIMART).

**Aim:**

To explore and describe the nurses’ experiences regarding operational factors influencing the implementation of HIV care services in the mobile health clinics (MHCs) of eThekwini Municipality in KwaZulu Natal.

**Methods:**

Qualitative Exploratory Descriptive (QED) method was used after permission was granted from North-West University Human Research Ethics Committee provincial and local health authorities. Data saturation informed sample size of thirteen MHCs nurses were purposefully sampled to participate. Audio-recorded, semi-structured, online, one-on-one interviews guided by open-ended questions were done for data collection, and including demographic profile. The interview transcripts were analysed using Atlas-TI and SPSS descriptive statistics was used for demographics.

**Results:**

Eleven subthemes emerged under patient-related, nurse-related, and organisational-related themes which influence the operational factors in the MHCs, namely: patient defaulting treatment, lack of privacy, unavailability of phones, stressful and demotivating MHCs, nurses feel unsafe, lack of support from management, lack of budget, unavailability of computers, shortage of medical equipment, shortage of nursing staff and absence of data capturers.

**Conclusion:**

Structured contextual coaching and support program for nurses is imperative to ensure effective and strengthened operations in MHCs, further supported by improvement in human resource for health allocation for MHCs in light of expanding health care programs

**Contributions:**

Evaluation of health care programmes, and human resource for health quality improvement needs in the clinical practice of HIV care of MHCs nurses which advocate for specific policy formulations.

## Background

Decentralized human immunodeficiency virus (HIV) care plays an important role in the HIV care continuum enforced in various care modalities including mobile health clinics (MHCs). MHCs are a community-based intervention that functions specifically as a delivery model for various medical services, including primary care and screening, preventative specialty care, and social interventions [1, 2]. Registered nurses in the South African public health system are the main drivers that operate the MHCs [3], which are also used as an additional healthcare delivery approach aimed at HIV program expansion, without misplacing their initial inception purpose of serving general populations with healthcare access challenges [4]. Instead MHCs have been officially acknowledged and accepted as primary health care (PHC) service extension for remote areas within the local district health system [2] offering various healthcare services including HIV care, which is the focus of this study.

Delivery of healthcare services via MHCs generally, is considered valuable, especially for the vulnerable and hard to reach communities as they experience the lowest access to healthcare [5], yet such communities display the greatest burden of disease [6]. MHCs are progressively used in both urban and rural settings, found either in low, middle and high income countries to deliver wide range of healthcare service packages [7]. However research suggests that MHCs are the untapped resources of the healthcare system [8]. The Advantages of MHCs have been highlighted by various studies [9] and they include serving the at-risk populations and promoting high-quality care at low cost. In SA such services are provided free of charge especially by the district health system. Furthermore these MHCs are known to render primary healthcare in a culturally competent way and function as a flexible model of healthcare delivery [10]. This positions the MHCs to be an essential component of the healthcare delivery system as affirmed in literature [11]. By taking health services to the community through MHCs, governments hope to intensify the use of these services and improve local people’s various health outcomes [12]. Furthermore, MHCs have been cited as playing a significant role towards elimination of inequality and contributing in attaining universal health coverage within the healthcare systems [13]. The Joint United Nations Programme on HIV/AIDS (UNAIDS) highlighted that MHCs can play vital strategy towards achieving universal access to health goal especially in Brazil, Russia, India, China and South Africa (BRICS) countries [14].

In the South African public healthcare system, the registered nurses are the main role players in the MHCs operations [15], although certain contexts may have a different setup [12]. The services which are provided by the nurses in MHCs are usually not far-fetched from those provided by local healthcare settings. In terms of HIV care, which is one of country’s priority areas [16], MHCs contribute substantially throughout the series of HIV care continuum. As people living with HIV (PLWH) attending MHCs services complete several steps along care continuum, consisting of HIV testing and diagnosis, linkage to and retention in primary HIV care, and receipt and adherence to antiretroviral therapy [17]. Successful attainment of certain HIV care indicators through MHCs have been reported [18], and are anticipated since nurses working there are expected to have been exposed to the nurse initiated management of antiretroviral therapy (NIMART) training and certification. NIMART training is a short course on essential HIV clinical management that tackles the fundamentals of HIV management and includes prevention, diagnosis, treatment initiation, management, control, referrals, monitoring and evaluation of treatment success [19]. It was specifically developed and aimed at registered nurses working in the primary health care (PHC) settings in order to improve access to antiretroviral therapy (ART) [20]. NIMART training was introduced in South Africa in 2010 [21]. Certification after training is awarded only upon successful completion of a portfolio of evidence (POE) containing a range of competencies, which includes various number of adult and children cases for initiation of ART and follow up and [22] in certain settings certification can be awarded following formal objective structured clinical examinations (OSCE) having taken place to assess nurses’ competencies [22]. So, rendering HIV competent care within the MHCs context is possible.

According to the researchers’ knowledge, no study has reported on the operational factors affecting the implementation of HIV care in the MHCs context. Operational factors in this study refers to all the activities that are involved in the running of the MHCs specific to HIV care. These HIV related activities are believed to be of essence because they will fit in with the MHC design, due to their unique flexible deployment nature which in most cases responds directly to the needs of the communities that they serve, and linking them to priority healthcare programmes [23]. Operational factors in the c context are deemed important as they can provide informed feedback, while facilitating process evaluation for offered HIV care services. Therefore, this study’s objective serves to disseminate experienced operational factors undertaken by registered nurses working in the MHCs of eThekwini Metro in KwaZulu Natal province of South Africa. .

## Research design and methods

### Study design

A Qualitative Explorative Descriptive (QED) design was employed to explore and describe the operational factors influencing the implementation of HIV care services in the MHCs of eThekwini Municipality in KZN. Advantages of using the QED research design is that it aids the researchers to fully understand the participants’ experiences in their real world in terms of what works and what is not [24]. Therefore, a detailed account of day-to-day events on operations in the MHCs were captured including their characteristics. Literature advocates that QED design is suitable in scenarios where not much is known about the phenomenon of interest as it is in the case with MHCs operations of the study context, or the problem is too complexed to be captured by other methods (e.g., questionnaire survey) [24]. The highly pragmatic nature of QED allows for answering of solid and realistic ‘what’ kinds of question [24], such as those addressed in this study.

### Study setting

The study setting was eThekwini Metropolitan District of KwaZulu Natal province in South Africa which consists of urban, and rural/ peri urban areas divided in Central, Northern, Southern and Western subdistricts [25]. Healthcare delivery throughout the Metro is via District Heath System (DHS) through 17 district hospitals, 8 community health centres and 233 primary healthcare clinics [25]. DHS emphasize the importance of organizing and coordinating health service delivery at the local level as the strategy embodies a decentralized, area-based, people-centred approach to health care [26]. A total of 36 MHCs teams are available within eThekwini District, and they are attached to one of the fixed health facilities mentioned above. Each MHCs team has multiple mobile points (locations) which they service, and those mobile points are unique from each other. Each mobile point is regarded as a standalone MHC. Each MHC team have between 5 to 20 MHC points they operateAccording to Statistics South Africa (Stat SA), eThekwini municipality was found to be home to 3,199,000 people as of mid-year of 2022, which is seen as an increase of 0.72% from the previous year [27]. Widely spoken language is isiZulu followed by English and the rest of other 11 official languages [28].

### Population, sample size, technique, and procedure

Registered nurses who work in the MHCs that provide HIV care related services constituted the study population. Each MHC team of 36 teams had a minimum of two registered nurses totalling 72 registered nurse eligible for study population. Thirteen registered nurses who were NIMART trained from various subdistricts within eThekwini District formed an appropriate sample size [29] which was guided by reaching data saturation [30, 31] and non-probability purposive sampling technique was used [32] to select the study participants in line with qualitative nature of the study Guest *et al.* (2006). Recruitment of participants took place through various methods including (a) schedule physical meetings which took place in the central MHC departure points usually in the mornings before they could go out to various locations, (b) written recruitment materials which were distributed prior and during the physical meeting and (c) electronically in a form of email to their MHCs management and human resource departments. 

### Inclusion and exclusion criteria

Only professional nurses who were NIMART trained were included in the study. Additionally, such nurses should have had a minimum of two years working experience in MHCs because they would provide in-depth knowledge about HIV related activities in that setting. Furthermore they needed to have had , access to a smartphone with WhatsApp and one gigabyte data bundles was to after supplied for interviews. WhatsApp was the possible means of communication since the study was conducted during when Covid-19 pandemic restricts were imposed.

### Data collection

Commencement of data collection took place after obtaining informed consent from all the participants. Semi-structured English interviews were done remotely using WhatsApp guided by researcher-developed interview guide [33]. WhatsApp was deemed suitable means of data collection method since Covid-19 lockdown restrictions were in place during data collection and had to be observed to mitigate against the spread and control of the infections. All the nurses had access to a smartphone which promoted recommended ‘social distant’ method [34] of collecting data in the midst of restrictions. Interviews took place through WhatsApp video calls, which lasted between 45 minutes to over an hour each, and they were all recorded. Flexibility in terms of location and time for data collection was observed and controlled by the participants availability and suitability as outlined in literature [35]. Transcription of all the recorded interviews took place shortly after each interview and data analysis took place thereafter.

### Data analysis

Inductive theme content analysis was embarked upon using computer assisted qualitative data analysis software (CAQDAS), precisely ATLAS.ti was deemed suitable, as it has been used extensively in healthcare studies [36, 37]. The following seven steps were followed: 1. Becoming familiar with the data, which commenced during data collection and continued through data transcription. The authors emersed themselves with data through in-depth reading. 2. Generating initial codes, by uploading all the project document on ATLAS.ti and organised according to the project order. This was initial coding which aimed at labelling the topics as they were mentioned by the participants as part of describing data 3. Building a coding frame, 4. Searching for themes, 5. Reviewing themes, 6. Defining and naming themes and 7. Producing the report. Throughout the analysis process a co-coder was assigned, and served as a validator of codes, themes, and sub-themes. Furthermore, descriptive statistics using statistical package for social science (SPPS) was used to analyse the demographic profile of the participants.

### Ethical considerations

Permission to conduct a study was approved by the following three entities: North-West University Health Research Ethics Committee (reference no: NWU 00934-19-A1), KwaZulu-Natal Province Department of Health (KZ_202002_017), and eThekwini Metropolitan Municipality Health Unit (30/1/1/6/3/1). All nurses participated in the voluntarily, and anonymity, confidentiality as well as privacy was maintained throughout, by ensuring that no names were attached to the nurses nor mobile clinic names during the interview process. Instead, a coding system was created to link the participants and only the research team had access to that data. Written consents were obtained from all the study participants, through the following process: an initial meeting was set up with various sub-district managers to explain the study, and once permission was granted then the registered nurses were approached as a second step whereby full explanation about the study was conducted including opportunity of asking questions and answers was provided. Thereafter, logistics on how and when data collection was to be conducted using electronic media was explained. Finally, online written consents were obtained through using Google Forms which were sent via WhatsApp or SMS and verbal consents were also obtained on the day of the interview.

### Trustworthiness

Four general principles of trustworthiness [38] were adhered to in the study. Member checking was used to ensure credibility during interviews [39]. Transferability was achieved through detailed description of the context. Dependability was achieved through providing detailed participants characteristics, including their methods of selection to the study, and account of all research steps and results were described in detailed [40] this process was embarked on by S.J.N & L.A.P who are study researchers. Peer debriefing was also used for dependability because it is a solid communication habits that create trust [39], peer researchers were requests to read and comment on the study data, furthermore the researcher also engaged in reflexive auditing. The first researcher S.J.N who is not the member of staff or the MHCs conducted the interviews, and she had no personal nor professional relationship with participants, that ensured confirmability for the study [40], furthermore confirmability was also achieved through study findings which were grounded in the data, using participants’ quotes, and validating the interpretations of the data with the study participants.

## Results

### Biographic profile of the participants

**Fig. 1:**
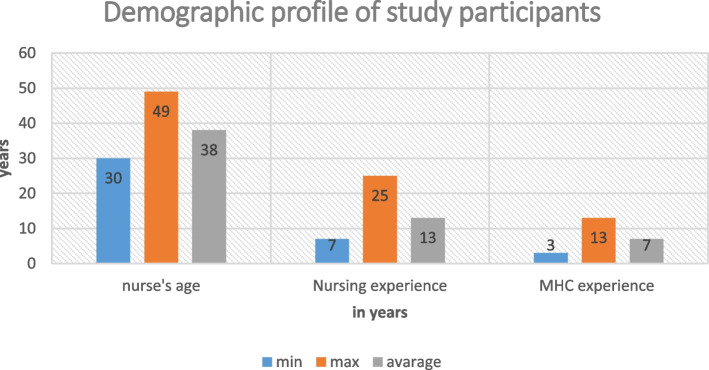
MHCs nurses’ age, experience in nursing and in MHCs environment

**Fig. 2:**
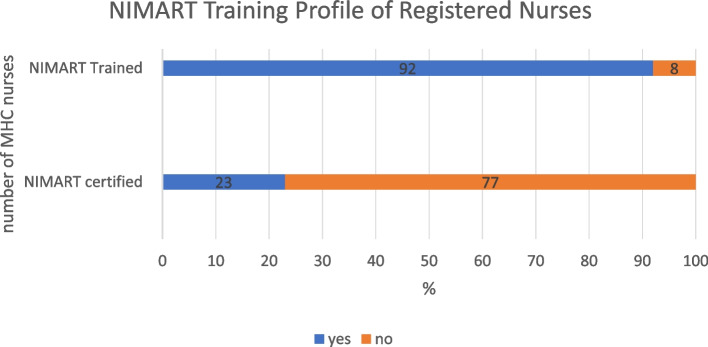
MHCs nurses’ NIMART training profile and certification rate

Registered nurses’ age ranged between 30 to 49 years, with seven to 25 years of clinical nursing experience and three to five years working in MHCs , as depicted in Fig. 1 below. Figure 2 contains details about NIMART training, certification, and the time that such training took place. . Majority 12 (92%) of the nurses had undergone NIMART training, only 3 (23%) received certification and training had taken place four years ago on average, however the rest of the training took place between one to nine years..

**Fig. 3:**
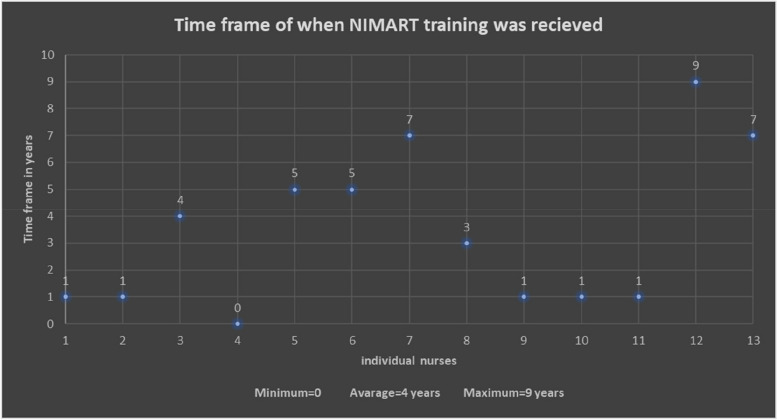
Time period NIMART training received by MHCs nurses

Training of MHCs nurses on NIMART is indicated in Fig. 3, precisely how long ago that training was received. The mean being four years and range being zero to nine years.

## Themes and subthemes

Three main themes emerged from the study denoting operational activities influencing implementation of HIV care services within the MHCs, which were related to patient, nurse, and organizational factors. Eleven subthemes also emerged related to various factors and are depicted in Table 1.

**Table 1:** Study themes and sub-themes

**Themes**	**Sub-themes**
1. Patient-related factors	1.1 Treatment dropout among patients
	1.2 Lack of privacy for the patients
2. Nurse-related factors	2.1 Nurse’s incapacitated means of communication
3. Organisational factors	3.1 MHCs are stressful and demotivating
	3.2 Nurses feel unsafe at MHCs
	3.3 Lack of support from management
	3.4 Lack of budget
	3.5 Unavailability of computers
	3.6 Shortage of medical equipment
	3.7 Shortage of nursing personnel
	3.8 Absence of data capturers

### Theme 1: patient related factors

Earlier studies have reported on patient factors as contributors to the factors influencing implementation of HIV care, but such reports never included MHCs contexts. Literature mostly reported factors from the urban versus rural community health clinics viewpoint [41].

#### Sub-theme 1: treatment dropout among patients

Patients are one of the main stakeholders in the healthcare system as they are the core consumers of the services rendered [42]. According to the South African patient’s right charter [43], patients have responsibility to comply with the healthcare system. However, reports from nurses in this study demonstrated that patients who seek services from the MHCs fail to comply with the healthcare system requirements, particularly those seeking HIV care. The patients go into the MHCs and pretend to be naïve HIV patients and lie to the nurses pretending to be new patients. Meanwhile they are known patients who have defaulted treatment elsewhere and due to fear of going back to where they receive their HIV care from, they resort coming to the MHCs under false pretence.

*“They* [patients] *come running to the mobile and pretend like they are new clients, meanwhile they had defaulted from the fixed clinic”* (Nurse 2, female, age 35)

*“…..we receive people that have been defaulting from the fixed clinics, and they come to us as if they are new. They just test* [for HIV] *as if it is the first time they’re testing. ”* (Nurse 1, female, age 35)

#### Subtheme 2. Lack of privacy for the patients

Privacy is the global term that suggests protection of the physical, dispositional, and informational dimension of an individual [44]. Privacy concerns in PLHIV have been reported before, but from the patient’s perspective [17]. In this study, lack of privacy has emerged from the nurses’ perspective, and it is perceived as a factor, affecting the daily operations in the MHCs.

*“How do you give services where there’s no privacy? I mean we work underneath the gazebo. Someone outside the gazebo hears everything happening inside”* (Nurse 7, male , age 40)

*“We do get a little bit of resistance from our patients whereby they don’t want to be initiated at the mobile points because of stigma as there is no privacy”* (Nurse 10, female, age 30)

### Theme 2: nurse-related factors

#### Subtheme 3: nurse’s incapacitated means of communication

Nurses highlighted that appropriate means of communication are not available at the MHCs. Yet communication in any healthcare setting is very important [45]. In the context of MHCs, communication is always needed with various stakeholders for an example, contacting the laboratory, line managers, community leaders and patients. If no means are provided for the nurses to be in contact with stakeholders, that impacts negatively on patient interventions since some of the MHCs only operate once a month in certain areas.

*“wherever we are there are no telephones as there’s no means for us to even stay connected with the fixed clinic, we are just alone. We used our phones to do most of the things, when you run out of something we use our phones even in cases of an emergencies”* (Nurse 11, female, age 42*)*

*“My dear there are no phones if you are in the crisis you use your own phone, and no one even bothers to compensate you. When I need to call an ambulance, police station, fixed clinic or even my manager I use my own phone”* (Nurse 3, female, age 36)

*“…we don’t have like phones sometimes we don’t even have the network when we are trying to phone”* (Nurse 12, female, age 47)

#### Subtheme 4: MHCs are stressful and demotivating.

Job satisfaction influences health worker motivation, staff retention and performance, which in turn impact on the successful implementation of health system reforms [46]. Literature reports that organizations and people that operate MHCs are often motivated by a commitment to underserved communities [47]. However, nurses in this study had a different mood about working in the MHCs.

*“I do not want to lie, I do not feel-good working here and seeing our poor people and being unable to provide them with care. it is not like you do not know what they need you know exactly but then you don’t have what is required. It is truly demotivating”* (Nurse 4, female, age 31)

*“...mind you already when you come you are dog tired from driving as we don’t have any drivers, and our* [mobile]*points are very far”.* (Nurse 6, female, age 32)

#### Subtheme 5: nurses feel unsafe at MHCs

Safety concerns of community nurses have been reported as they travel within various communities [48], even nurses in this study reported the same concerns.

*“One does not feel safe at all in the mobile points and once all the patients are gone, you become a target, mainly because of the* 4x4 [van that is equipped with four-wheel drive (4WD)] *that you are driving”* (Nurse 1,female, age 35)

*“we are not protected in the mobiles, I mean the communities that we visit sometimes are not safe and being in possession of ARVs puts us in danger because they stop us demanding these ARVs on the road apparently”* (Nurse 2, female, age 35)

### Theme 3: organizational factors

#### Subtheme 6: lack of support from management

It has been proven that inadequate management and poor leadership roles lead to failure in healthcare delivery system [49] and leads to job related stress [50, 51].

*“There is no support from our managers for the mobiles if we can be honest. Mobiles are expected to function exactly like the fixed facilities, yet they* [managers] *are never involved in the programs or activities that are being run”* (Nurse 8, female, age 47)

*“There’s no support at all. Gosh. There is no support. It’s so frustrating. You want to try, and you know what, sometimes……. It’s just impossible because you can’t do it alone!”* (Nurse 1, female, age 35)

#### Subtheme 7: lack of budget

For a successful implementation of any healthcare programme, a budget needs to be put in place, well before programme activities can resume. However, seemingly in relation to MHCs operations such budget does not exist. However, this is not unique to MHCs but instead a study in South Africa [52] found during an audit that, none of PHC clinics were fully or adequately budgeted for. It is therefore not surprising for MHCs to report budget issues since MHCs are an extension of PHC clinics.

*But if you raise the issue of staffing with the managers, they tell you there’s no budget. So, if there’s no budget, they also need to accept that there are no proper services* (Nurse 4, female, age 31*)*

*“We constantly have to depend on the left over from the fixed clinics, we don’t have standalone budget dedicated to us as mobiles”* (Nurse 7, male, age 40)

#### Subtheme 8: unavailability of computers

All MHCs activities need to be captured and entered on the District Health Information System (DHIS) [53], to measure and evaluate some key health indicators. HIV care elements are no exception, especially data on HIV positive patients remaining on ART (TROA). However, nurses reported that the data capturing activities are problematic at the MHCs sites due to lack of working tools such as computers, which are essential for capturing and feeding of information through to the district office.

*“They* [patients]*default from the clinic knowing that we wouldn’t know, because we don’t have even the computer to check them on the system”* (Nurse 1, female, age 35)

*“We don’t have a computer to actually capture this people for tier. So, those are the challenges”* (Nurse 5,female, age 34)

Currently data at MHCs is handwritten in registers by nurses and aggregated into data sheets for submission to the District office which is similar to what has been reported previously [53] . Problems with this approach include a high work burden and a lack of training as perceived by the nurses, as well as poor data quality [54]. This acts as a barrier for the effective running of the service and concurs with the findings of [52] where clinics were not adequately prepared to render integrated HIV services. However it is appreciated that its could be difficult to carry desktops around in the MHCs because theres are no physical structures in some of the MHCs points no eletricity. But a laptop or a tablet with built in software will surfice.

#### Subtheme 9: shortage of medical equipment

Nurses depend on functional medical equipment in order to perform their duties which include prevention, diagnosing, monitoring and treating diseases not excluding rehabilitation activities [55], Therefore medical equipment is at the core of delivering effective healthcare. Their shortage impedes on effective rendering of services, desolately MHCs nurses expressed a great challenge over medical equipment availability.

*“For instance, right now we don’t have a scale, its broken because each day we have to go up and down with in the car and sometimes the roads we travel in with other points its soooo* [*e*xtremely] *rough”* (Nurse 3, female, age 36)

*“We don’t have the basic tools or equipment that are needed so that we can function well and provide HIV services as how the managers expect us to do”* (Nurse 2, female, age 35)

*“I mean it doesn’t help to blame the nurses saying they are not providing the service meanwhile they are not given the necessary tools and equipment needed to complete such a service”* (Nurse 5 female, age 34)

Unfortunately the shortage of medical equipment phenomenon is reported to be a widespread problem in various South African healthcare contexts [56, 57], hence no surprise when the MHCs nurses report of it as well. Researchers in this study agree with [58] who stated that serious health care repair is required, as research conducted in fixed clinics revealed that none of the clinics were adequately equipped [52].

#### Subtheme 10: shortage of nursing personnel

Inadequate of human resource in healthcare is not a new phenomenon even in the global arena [59], and it has been reported as being worse in sub-Saharan Africa and is a major weakness in healthcare system [57] attributed by various reasons [57]. Regrettably, further predictions have been made by the World Health Organization on the problem worsening by 2035 [60]. A similar report emerged from this study

*“How can you run a mobile clinic alone* [being the only one NIMART trained in the team]*and looking after people who are sick, sick, sick? So, it’s a recipe for a disaster”* (Nurse 4, female, age 31)

*“I am not just one person. Here I am a driver, I am a cleaner, I am a clerk, and I am a nurse”* (Nurse 4, female, age 31)

Attending to patients’ seeking HIV care require time in order each individual needs of the patients can be attended to fully. But if these patients are seen at the MHCs which only have a single professional nurse who still needs to attend to other patients-this will results in lessen time spent on the patient which might affect the overall quality that is received at the end of the day. Therefore, additional nursing trained and certified in NIMART is highly recommended in order to improve effectiveness.

#### Subtheme 11: absence of data capturers

Data capturing of clinic service is of essence since all operation decisions are made based of the information available. Therefore, the source of data is equally important as its use. Hence ensuring that data in captured timeously and accurately by someone trained to do it, is important. However, nurses in the MHCs report that there are no data capturers.

*“We don’t even have a clerk who could be doing this.* [it is] *time consuming in short and very much delaying ”* (Nurse 4, female, age 31)

This is not only unique to MHCs as it was reported before in the PHC settings [46]. The lack of instrumental HIV care staff such as data capturers results in increased nurses workload, while increasing the risk of doubting the integrity of captured data due to the fact that only one professional nurse is working there and not necessarily trained to be data captures [53], in the midst of existing challenges. Furthermore, some of the nurses are not even tech no savvy. A need for innovative ideas to capture data smartly should be viewed as urgent. Alternative methods such as mhealth in MHCs would be considered as they possess great benefits [61] such as the availability of smart phones is widespread to be operated by a designate person for a task.The wealth of data collected across the MHCs needs to be accurate, consistent and appropriate, to provide timely and reliable information which can be viewed intergrately reporting, measurement and analytics needs [62]. Futhermore the MHCs also need to implement the systems that are adopted by the rest of the country. TIER.Net which which is a patient monitoring system for HIV patient visits was introduced in South Africa in 2010 [63].

## Discussion

Various existing gaps found within the MHCs have caused the stakeholders to take advantage of the deficiencies for their benefit. to capitilise for their advantage as no tracking system is in place. Defaulted treatment patients from other facilities present themselves to the MHCs as new patients, which leads to wasteful expenditure on already stretched supplies and compromised staff compliment in the MHCs. Furthermore other patients also get impacted in terms of waiting periods. Therefore if HIV care services are offered in the MHCs, they should be offered comprehensively with all its supporting systems required in place, which will prevent dublication of service and provide continuity of care. However MHCs contribute indirectly towards resolving the challenge reported [64, 65]of high HIV treatment defaulter rate in primary health care settings as patients get reorientated back to the healthcare system through MHCS. An urgent need to evaluate current MHCs’ infratracture and design is critical inorder to ascetain the feasbility of HIV care offereing through MHCs, because the results emintated from this study indicate that indeed current multi challenges existHuman rights should be central in any type of healthcare service that is provided irrespective of its setting. In this study pronounced lack of privacy was prominent regardless that such has been reported in the fixed clinics as well [41]. That has a potential to cause low service update [66, 67] due to indignitied and compromised care. MHCs should have innovative means of communications in place since they are rendering care in secluded and remote areas. His study highligligted how the importance of communication was neglected completely and nurses had no means of contacting the outside healthcare departments once they are outside in the MHCs yet continuation of care and followup could be made easy through effective communication methods. On the hindsight nurses in the study resorted in using their personal communication devices without compensation in order to fulfil their professional duties. Yet mobile devices usage in healthcare have been endorsed for over two decades already [45, 68, 69]. The communication challenge findings in this study are similar to what has been reported previously [45].

The degree of stress reported by nurses in this study is concerning since there direct correlation between work satisfaction and the quality [70] produced. Duties performed under stress are easily prone to professional errors [70], thereby impacting of the quality of HIV care rendered at the end.

Danger associated with working in the MHCs has been reported in terms of personal, physical, and psychological safety, attributed by lack of security personnel escorting them in some MHCs points, female dominated profession [71] whereby they are easily taken advantage of based on their gender. Also carrying ARVs with them which are used for formulation of illicit drug whoonga [72], which is linked to criminal activities [72] following its consumption. This could be very conflicting for the nurses as they are bringing much needed drugs yet fall victims of crime because of the very same drugs. Balancing own safety and that of patient has been document in literature as one of the gray areas [73], such as the situation MHC nurses find themselves in.

With all the stated challenges noted in the study findings, a possible solution could be reached whereby management at operational level could be harnessed through visible and intentional management of the MHCs. Current status reported by nurses stated that lack of support and supervision is prevalent, which is similar to what has been reported before [41]. Unfortunately, such conditions will yield to unfavourable programmatic outcomes as evidenced in this study. Therefore, managers’ support in any format is deemed crucial, even more in settings such as MHCs. The gross lack of support cited in this study is contrary to other study [74] where nurses and their managers felt optimistic about HIV care and had increased job satisfaction. The absent support demonstrates no good working partnership between the managers and their subordinates and literature [75] describes this as a complex problem where unsupportive relationships within their work group exist and always results in poor outcomes. Lack of support therefore paralyses the MHCs nurses not to even exercise their contextual innovative capabilities as no one could endorse them. But instead, they are expected to do things by the book which sometimes are challenging to be implemented in the MHCs context, as in most cases such policies are formulated and designed for the fixed clinic environments which are different from MHCs. . Also, these factors will impact negatively to the overall HIV care rendered by MHCs nurses. Tangible presence, guidance, affirmation, and mentorship in the MHCs by their managers should be prioritised, to keep the nurses motivated while monitoring ongoing monitoring and evaluation of HIV care services. Once this if fulfilled then MHCs will function effectively to a certain extent.

## Strenghts and limitations

The qualitative nature of the study was able to capture the voices of the nurses that are rendinring HIV care in the MHCs context and a nature of the study has never been conducted before. Theirfore the study findings forms the basis for further research and can influence policies in terms of how management and operations are done in the MHCs.

Limitations of the study include the fact that the study was only conducted in one district in KwaZulu Natal, therefore the results cannot be generalisable to all the MHCs in the province, national, continental and internationally.

## Implications of the study

The study demonstrated a great need for mobile health services to be entrenched on the six health systems building blocks outlined by the WHO health system framework 2007 [76] i.e. service delivery, health workforce, information, medical products, vaccines and technologies, financing and leadership/ governance mentorship. Unfortunately, all the building blocks have shortcomings. Following few suggestions can be considered such as a structured contextual coaching and support programme should be in place, and to be led by nurse managers, while enhancing and promoting peer buddy mentorship system.

Operations would be better enhanced if the following practical suggestions are effected: support and staff provision, introduction and accurate use of district information system, specific budget allocation, incentive consideration, and recognition that MHCs cannot operate the same as the fixed PHCs.

## Conclusion

This study demonstrated a serious gap that exist and the challenges that the nurses in MHCs are faced with day in and day out without any support whatsoever. Priority consideration and attention regarding the MHC operations is urgently needed since this study highlight the importance of HIV care delivery through the MHCs while in the presence of challenges. Reflections on the quality of HIV care service offered to the community members should be conducted in line with human rights. While assessing if they are being met for both the patients and the nurses who are in operation of these clinics. Indirectly the MHCs mimic what the local PHC clinics look like in terms of their operations as in most cases within the district MHCs are attached to either CHC or PHC establishment.

## Recommendations

Policy gaps in health delivery in MHCs are significant and should be looked at with specific lenses addressing contexual issues by those in leadership and strong governance should be implemented inorder to improve and upscalling of services provided in the MHCs. The entire of MHCs needs to go through some reformation inorder to meet the current health needs, whereby infrastructure that will respond to the needs eg for both the patients and the staff working there eg patient privacy, basic necessary tools that aid in perfomance of nurses’ duties. could be implemented. Furthermore, attractive incentive packages should be offered to MHCs nurses, such incentives have a potential to increase nurses’ morale, decrease attrition rate, and attract more nurses to work in the MHCs Platforms for debriefing, pschological supports and incentives would form examples of what is needed including focused area capacity buiding activities which would be inclusive of mentorship. Stimulation of MHCs nurse’s ethics should be stimulated inorder for their automonony over services and the environment (conditions) they are working in. A system to ensure that all the MHC patients are entered in the data based is critical, even if it means such patients are inputed under the fixed clinic that the MHCs are attached to. Such access will enable patients’ unique identifers to be dipicted as well as facilitates person-centred monitoring as per WHO guidelines. This would be possible because MHCs personel report to the fixed clinic or central office everyday before and after every MHCs visit. Due to existing infrastructural challenges as lack of eletricity and proper office supplie in the MHCs, potable monitoring devices in a form of tables to capture esssential data can be introduced, devices similar to the ones that are being used during census.to serve such a purpose. Furthermore, to adress identified shaortages of staff, MHCs stall complement should be increased to atleaste 3 professional nurses per MHC with another trained member of staff who will be responsible for data control.

Legislative framework applicable to workplace not limited to Basic Conditions of Employment Act, the Occupational Health and safety Act, Nursing Act and Policy on Nurses Rights should be incoparated significantly in the MHCs work environment. Impact studies need to be conducted to evaluate the need for the services.

## Data Availability

The dataset used and analyzed during the current study is available from the corresponding author on reasonable request.
